# Xanthogranulomatous Pyelonephritis in a Young Adult Without Any Pre-existing Conditions

**DOI:** 10.7759/cureus.63914

**Published:** 2024-07-05

**Authors:** Jerin Varghese, Abhijit Dhale

**Affiliations:** 1 Department of Urology, Datta Meghe Institute of Higher Education and Research, Wardha, IND

**Keywords:** symptom, xgp, kidney, pyelonephritis, xanthogranulomatous

## Abstract

The timely diagnosis of xanthogranulomatous pyelonephritis (XGP), a rare and chronic kidney condition, along with its appropriate management, is a must to spare the kidney from end-stage renal disease (ESRD). The main hurdle in early diagnosis of most medical conditions, including XGP, is the absence of specific and characteristic symptoms, which, if present, would make the patient seek medical aid earlier and tempt the clinician to think of variable differential diagnosis. We hereby report a case of a 20-year-old male patient who had no specific symptoms suggestive of a renal pathology, which delayed him from considering consulting a healthcare professional, thereby making his condition diagnosed as XGP at a time when his involved kidney was hardly salvageable. Through this case report, we wish to humbly request clinicians all across the globe to kindly broaden their range of differential diagnoses while dealing with patients with nonspecific symptomatology, in order to have a better prognosis.

## Introduction

Xanthogranulomatous pyelonephritis (XGP) is a rare (0.6% to 1% of all renal infection cases) variant of chronic pyelonephritis, which is aggressive, ultimately landing the kidney into a non-functioning state [1,2]. It is a destructive granulomatous inflammation of the renal parenchyma [3]. XGP can be classified into diffuse, segmental, and focal XGP, with kidney involvement being diffuse, segmental, and within the cortex. The exact aetiology of XGP is still not known. Many assumptions have been made over time regarding the cause of this condition. Chronic urinary obstruction and infection are found to be the culprits in most cases. Various organisms contribute to the causation of XGP, including *Escherichia coli*, *Klebsiella*, *Proteus mirabilis*, *Enterococcus faecalis*, and *Pseudomonas* [4]. Different risk factors are associated with XGP, such as diabetes mellitus, hypertension, renal transplant, abnormal lipid metabolism, and immunocompromised individuals.

XGP often gets confused with renal cell carcinoma because of the similarities these two share with respect to clinical and radiological features, and their ability to involve organs and structures adjacent to them. Thus, confirming the diagnosis becomes a priority before heading to the management. Urine investigations in more than half of the cases do reveal pyuria [5]. Urine culture often shows the presence of multiple organisms. Blood investigations usually reveal leukocytosis, increased erythrocyte sedimentation rate (ESR), and anaemia. Raised aspartate aminotransferase and alkaline phosphatase, decreased albumin, and high fasting blood sugar are other laboratory findings that could be additionally present [6].

In diffuse XGP, ultrasonography (USG) typically demonstrates the affected kidney to be enlarged with a large amorphous central echogenicity corresponding to multiple fluid-filled masses, renal pelvis staghorn calculus, and pelvic contracture. Ultrasound findings in cases of focal XGP are non-specific and thus make differentiation from similar conditions practically impossible [7]. Computed tomography (CT) scans, over the years, have emerged as the most trustworthy and sensitive investigation to confirm the diagnosis of XGP. The increased availability and usage of CT over the recent years have brought many XGP cases into light that otherwise would have never been diagnosed if there was limited use of this valuable radiological modality.

Diffuse XGP kidney typically is enlarged with pelvic calculi, hydronephrosis, or some other conditions such as congenital type radiation fibrosis, obstruction, and ureteric carcinoma. Nodules that could mimic nodules of the tumour may be single or multiple with yellow to orange colour. Granulomatous mixed inflammatory infiltrate with fibrosis and cholesterol clefts in the background are characteristics of XGP [4]. Fine-needle aspiration cytology (FNAC) of XGP is characterized by individual or small clusters of xanthomatous cells, and cells showing a glandlike pattern that might be from degenerative renal tubules are the characteristic features of FNAC in cases of XGP [8].

Nephrectomy is the preferred treatment modality for diffuse or advanced-stage disease [9]. Antibiotics are helpful to a certain extent in acute exacerbations, but ultimately, the management would end up in a nephrectomy of the involved kidney. Symptomatic cases are diagnosed earlier if proper investigations are carried out timely and thus have a better prognosis than asymptomatic cases, which often are diagnosed at a very late stage when nothing much can be done other than nephrectomy.

## Case presentation

A 20-year-old male patient came to the Medicine Outpatient Department (OPD) with chief complaints of fever and associated cough for four days. The fever was insidious in onset, gradually progressive, relapsing type, with multiple episodes, no aggravating factors, relieved on medication (paracetamol), no diurnal variation, and associated with a nonproductive cough. The patient consumed a mixed diet, sleep and appetite were unaltered, and bowel and bladder habits were regular. There was no history of any addictions, and no history of any high-risk behaviour was present. There was no history of any serious medical or surgical illness, nor had anyone in his family experienced similar complaints.

On general examination, he was conscious, cooperative, and well oriented to time, place, and person; febrile, temperature was 100.1°F; pulse was 96 beats per minute on right radial artery, regular rhythm, normal volume with no abnormal character; respiratory rate was 20 cycles per minute, abdomino-thoracic; blood pressure was 120/76 mmHg on right upper arm sitting position. Pallor, icterus, clubbing, cyanosis, lymphadenopathy, and oedema feet were not present. Jugular venous pressure (JVP) was not raised. A systemic examination was also carried out. During the central nervous system (CNS) examination, all the cranial nerves were intact. On cardiovascular system (CVS) examination, S1 and S2 were heard; no murmurs were present. Bilateral air entry was equal on respiratory system (RS) examination; no abnormal breath sounds were present. On per abdominal (PA) examination, the abdomen was soft and non-tender; no evidence of organomegaly was found.

Investigations that were advised included routine blood and urine workups. Blood and urine samples were sent for investigation. Meanwhile, empirical antibiotics, antipyretics, and cough syrup were started, which helped alleviate fever and cough, and plenty of pus cells per high power field in urine and slight derangements in certain blood parameters were revealed. The blood investigation report is presented in Table 1. The patient's urine was then sent for culture, whose report later indicated the presence of multiple organisms.

**Table 1:** Blood investigation report Hb%: Haemoglobin percentage; MCHC: Mean corpuscular haemoglobin concentration; MCV: Mean corpuscular volume; MCH: Mean corpuscular haemoglobin; RBC: Red blood cells; WBC: White blood cells; HCT: Haematocrit; RDW: Red blood cell distribution width

Investigation date and time	Hb% (g/dL)	MCHC (g/dL)	MCV (fL)	MCH (pg)	Total RBC count (millions/mm^3^)	Total WBC count (cmm)	Total platelet count (x 10^11^/unit)	HCT (%)	Monocytes (%)	Granulocytes (%)	Lymphocytes (%)	RDW (%)	Eosinophils (%)	Basophils (%)
April 4, 2023; 3:25	7.8	33.6	83.1	27.9	2.79	6200	4	23.2	3	55	40	17	2	0
April 10, 2023; 4:09	9	32.8	83.9	27.5	3.27	6500	4.6	27.5	3	55	40	17.6	2	0
April 14, 2023; 2:19	9.6	32.4	84	27.2	3.53	5700	4.4	29.7	3	60	35	17.9	2	0

The patient was referred to the Urology Department, where he was advised USG of the abdomen, which showed suspicion regarding the right kidney as 17 mm hyperechoic calculus was noted in the renal pelvis of the right kidney, causing gross dilation of the pelvicalyceal system (PCS) with thinning of cortex (upper pole 9.2 mm, middle 5.5 mm, lower 7.2 mm). Signs of grade IV hydronephrosis were present. The left kidney appears bulky with an altered echoic texture, showing few hyperechoic areas. Kidney, ureter, and bladder (KUB) intravenous pyelogram (IVP) was performed. Further, he was advised to have a CT scan for a better understanding of the kidney pathology. CT urography was performed, which gave the impression that there is a non-excreting right kidney with obstructive pelvic ureteric junction calculus causing grade IV hydronephrosis and perinephric periureteric fat stranding, which is most probably as a s/o right-sided XGP. A renal diethylenetriamine pentaacetic acid (DTPA) scan (renal scintigraphy) was also performed, which showed poor functioning of the right kidney as it contributed just 2.9% of 75.6 mL/min of total glomerular filtration rate (GFR).

**Figure 1:**
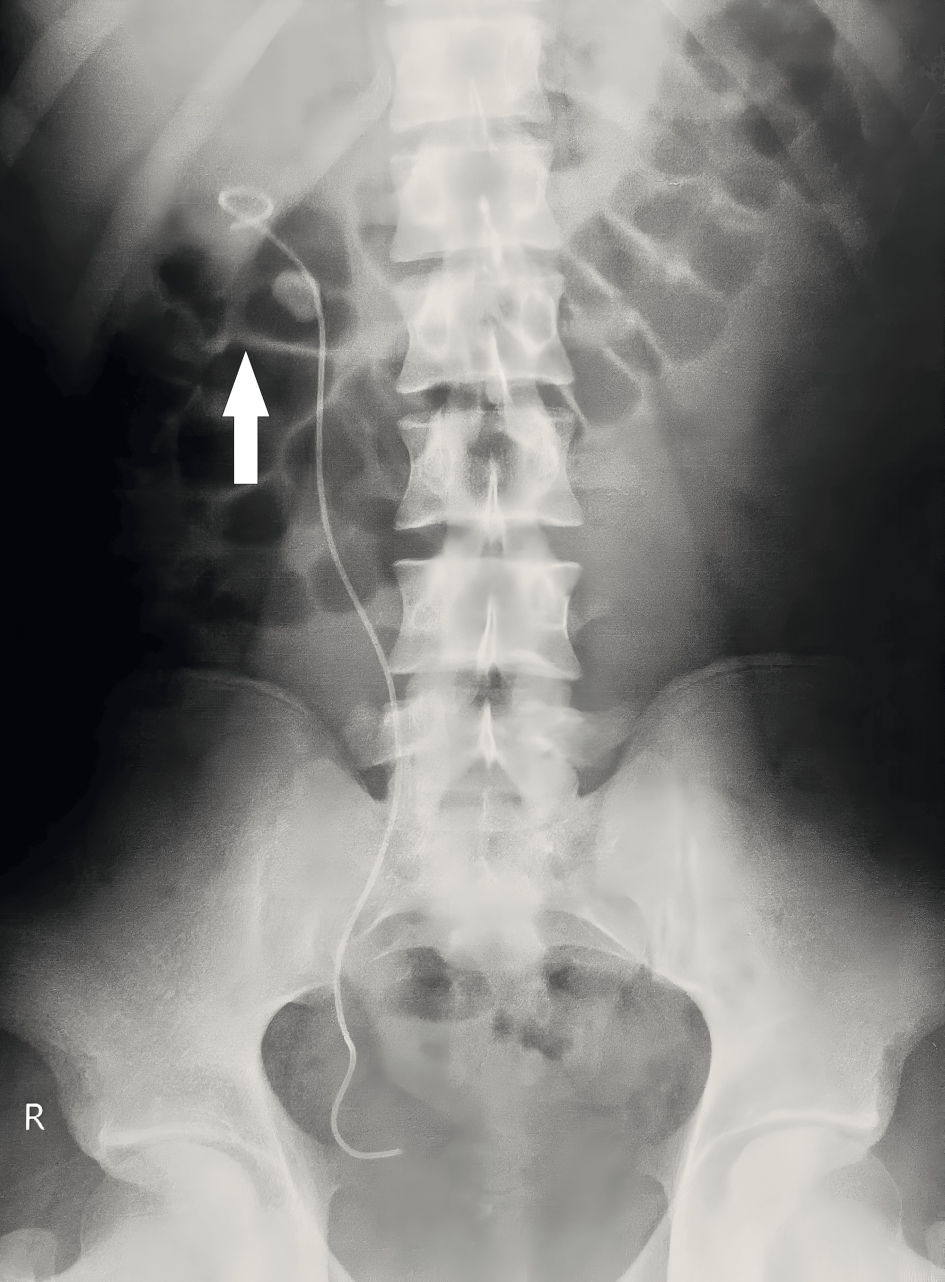
Radiological image after DJ ureteral stenting DJ: Double-J

Under all aseptic precautions and with the patient in a lithotomy position, painting and draping were done. Cystoscopy was performed with a 17 Fr. 30° telescope. Evidence of frank pus from the bladder was seen, which was drained out. Bladder wash was given with normal saline. Both the ureteric orifices could not be visualized, and thus the procedure was abandoned, and the patient was shifted to the recovery room for further management. Later on, the right double-J ureteral (DJ) stenting was carried out, and the imaging after the procedure has been put forth in Figure 1. These led to the establishment of the diagnosis of XGP.

**Figure 2:**
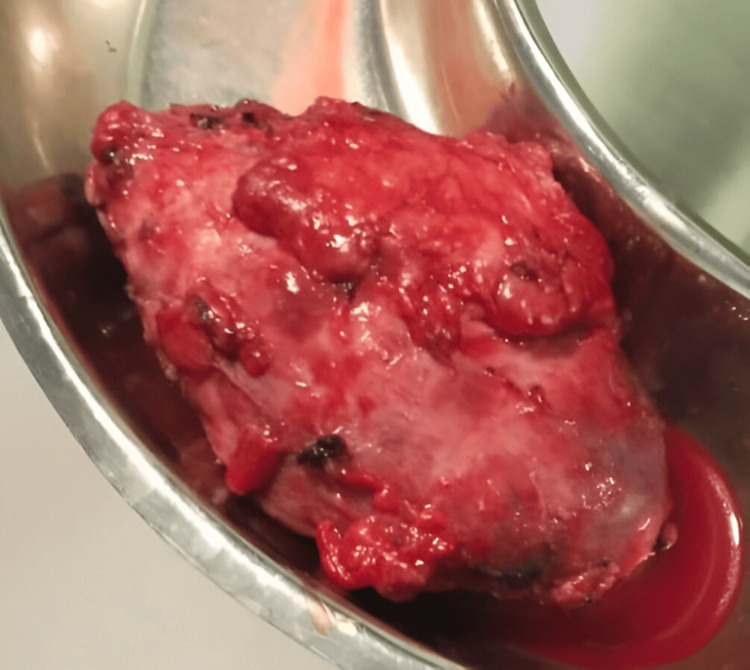
Nephrectomy specimen

**Figure 3:**
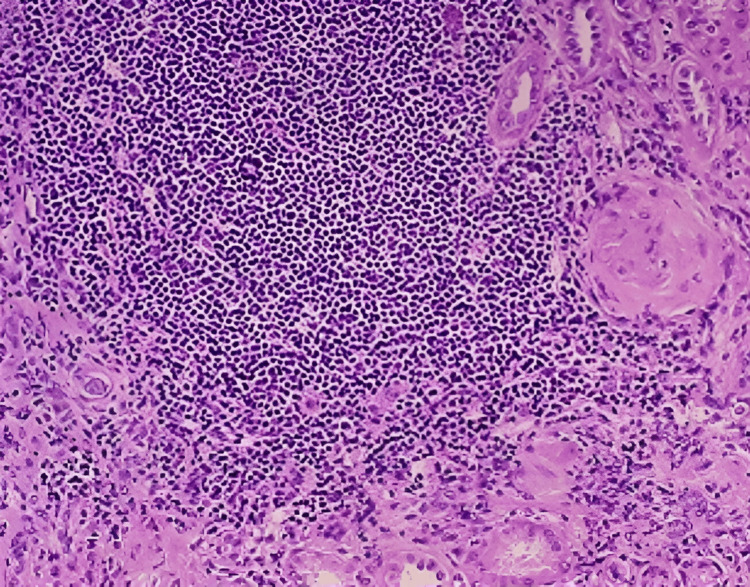
Histopathological image

The patient was informed about the condition of his right kidney. After proper explanation and counselling, the patient's consent was obtained, and a right-sided nephrectomy was planned to be carried out at a later date. Meanwhile, the patient was advised to consume plenty of oral fluids, and it was explained that the catheter drain continues to be kept in situ for drainage. He was then discharged. After two weeks, the patient was readmitted for right-sided nephrectomy. After obtaining surgery fitness, under all aseptic precautions, general and epidural anaesthesia was given. The patient was placed in the lateral decubitus position at a 45° angle with the lower leg flexed 90° and the upper leg extended. The kidney rest was elevated, and the table flexed and adjusted horizontally to obtain optimal flank exposure. Right subcoastal 11th rib cutting incision was taken. Dissection was done in layers; the 11th rib was dissected and cut. The peritoneum was pushed medially. The kidney was identified and separated all around from the duodenum and liver. There was evidence of a large lobulated right kidney with a stone in the upper ureter. The ureter was identified and looped. The right renal artery and vein were identified, dissected, and ligated. The ureter was then dissected and ligated. The specimen, whose image is enclosed in Figure 2, was removed and sent for a histopathological examination (HPE), which revealed findings suggestive of chronic pyelonephritis; the histopathological image is presented in Figure 3. Haemostasis was achieved and confirmed. A size 24 abdominal drain was inserted and secured. Closure was performed in layers using Vicryl No. 1 and Vicryl 2-0 round body sutures. The skin was closed using skin staplers. Sterile dressing was done. The procedure was uneventful, and the patient was shifted to the Intensive Care Unit (ICU) for observation. Later on, he was moved to the recovery room for further management.

Postoperative medications included cefoperazone, sulbactam, metronidazole, pantoprazole, ondansetron, paracetamol, and tramadol. The postoperative stay was uneventful, and thus the patient was allowed discharge. On discharge, the patient was advised to take a high-protein diet and plenty of oral fluids; proper hygiene was advised. He was called after two weeks to remove staplers, and he was informed to reach out immediately in case of any emergency. When the patient came for the removal of staplers two weeks after the operation, his condition was clinically assessed. His general health condition was good, and he had no complaints. He was able to resume his day-to-day activities. He was advised to report immediately if he ever had any issues in the near future.

## Discussion

XGP is more common in females than in men. Korkes et al., based on their experience with 41 cases of XGP, reported that 85.4% were women and 14.6% were men [10]. Usually, XGPs are present in the fifth and sixth decades of life, with their presentation in children being rare. XGP is typically a condition that affects unilateral kidneys, but there is no rule of thumb that it doesn't affect bilateral kidneys simultaneously.

Schlagenhaufer et al., in a series, reported that all the patients were symptomatic, and mostly these patients had more than one symptom; the typical symptoms included pain in the flanks or abdomen, palpable mass, symptoms of the lower urinary tract, fever, weight loss, gross haematuria, etc. [4,11-14]. Al-Ghazo et al., after analysis of 18 cases of XGP, came up with the results that calculi or obstruction in the urinary tract, kidney damage, urinary tract infection (UTI) complications, anaemia, liver dysfunction, and increased ESR were the characteristics of the condition clinically, with all the patients being of diffuse XGP; one-third of the patients had nephrocutaneous fistula, psoas abscess, paranephric abscess, renocolic fistula as associated pathological findings [5]. Ischemic colitis secondary to XGP was reported by Su et al., while Wen and Chen reported XGP complicated by emphysematous pyelonephritis in a haemodialysis patient [15,16].

Taking the above studies and facts into consideration, this particular case of a male in his 20s, having no flank or abdominal pain, no haematuria, no associated urinary symptoms (difficulty passing urine, burning micturition, etc.), with the only symptom present being on and off episodes of fever and cough, in itself becomes a rare presentation of XGP. Due to the very vague and non-specific symptoms experienced by the patient, he didn't seek medical help for around two weeks after the first episode of fever and cough. This case becomes even more interesting when one comes to acknowledge the fact that even till the day of the right-sided nephrectomy that he underwent, he didn't experience any pain at all.

It was the pyuria which raised the suspicion of renal pathology. Otherwise, from the patient's clinical presentation, the diagnosis of renal pathology was way too far. The USG report showed discrepancies, but as it was not specific, the patient was advised to undergo a CT scan, which confirmed more or less the diagnosis of XGP. The diagnosis was established better and more strongly by the urine culture reports, which showed the presence of organisms in them in considerable numbers.

The entire course of the disease clinically lasted for no more than two months ago from the day of nephrectomy, but the disease might have been in a dormant phase way back than this time period. Ironically, due to delayed presentation and generalized symptoms, the condition was diagnosed at a time when the kidney involved could not be salvaged. Once the patient sought medical aid, from there on, the patient was taken care of by an experienced multi-disciplinary team of doctors, but even after great efforts from the medical fraternity, due to the delayed presentation of the patient to the medical setup, his right kidney had to be removed as that was the only resort left.

## Conclusions

A male patient losing one of his kidneys at a very young age to XGP due to nonspecific symptoms is reported. The peculiar absence of flank or abdominal pain, along with no symptoms of renal pathology as such, is a matter of concern, as presentations like this case would delay the patient seeking medical aid, thereby leading to poor prognosis, as the diagnosis is confirmed at a point when any intervention could hardly spare the kidney involved. The reporting of such cases is the need of the hour in order to raise suspicion in the mind of the clinician, so as to make earlier diagnosis and hence appropriate management.
